# B-cell response in solid organ transplantation

**DOI:** 10.3389/fimmu.2022.895157

**Published:** 2022-08-09

**Authors:** Stephanie G. Yi, Ahmed Osama Gaber, Wenhao Chen

**Affiliations:** ^1^ Division of Transplantation, Department of Surgery, Houston Methodist Hospital, Houston, TX, United States; ^2^ Division of Transplant Immunology, Houston Methodist Research Institute, Houston Methodist Hospital, Houston, TX, United States

**Keywords:** B cells, alloimmunity, transcriptional (regulation), transplant, rejection

## Abstract

The transcriptional regulation of B-cell response to antigen stimulation is complex and involves an intricate network of dynamic signals from cytokines and transcription factors propagated from T-cell interaction. Long-term alloimmunity, in the setting of organ transplantation, is dependent on this B-cell response, which does not appear to be halted by current immunosuppressive regimens which are targeted at T cells. There is emerging evidence that shows that B cells have a diverse response to solid organ transplantation that extends beyond plasma cell antibody production. In this review, we discuss the mechanistic pathways of B-cell activation and differentiation as they relate to the transcriptional regulation of germinal center B cells, plasma cells, and memory B cells in the setting of solid organ transplantation.

## Introduction

Despite an overall improvement in 1-year graft survival (1), median kidney allograft survival is 94.3% at 1 year but decreases to 76.3% at 5 years (1, 2). Approximately 40% of kidney allografts fail in 10 years, and nearly 67% of failed allografts are due to T-cell-mediated (TCMR) and/or antibody-medicated rejection (ABMR) (3). Chronic ABMR has been identified as the leading cause of graft loss in kidney transplantation and appears minimally responsive to current immunosuppressive therapies (4). With inconclusive data from clinical trials (5–7), there is currently no standard of care for the treatment of chronic ABMR.

B cells play a key role in the long-term detrimental effect of alloimmune-mediated injury (8). In transplantation, B cells can produce donor-specific antibodies (DSAs). These antibodies promote acute and chronic rejection by activating complement which causes vascular injury and allograft loss. However, there are other effector mechanisms from antibody binding that contribute to allograft destruction. Human leukocyte antigen (HLA) (9) antibodies can have a direct effect on endothelial cell binding *via* Fc receptors on immune cells such as natural killer (NK) cells, macrophages, and neutrophils to mediate allograft injury (10). This mechanism of inflammation occurs independently of traditional HLA-associated complement activation (11), hence explaining why complement inhibitors alone are not effective against AMR (12, 13). There are also anti-HLA antibodies that are directed toward alleles not found in the recipient (14). Increasing evidence suggests that these pathogenic antibodies can be directed at minor antigens and autoantigens in the transplanted allograft (14, 15).

The transcriptional regulation of B-cell response to antigen stimulation is complex and involves an intricate network of dynamic intra- and extracellular signals from cytokines and transcription factors. There is a strong interest in understanding B-cell immunobiology as it relates to antibody development and production in response to solid organ transplantation. B-cell contribution to alloimmunity ranges from plasma cell (PC) differentiation and maintenance of long-term humoral immunity, serving as antigen-presenting cells, organizing the formation of tertiary lymphoid organs, and secreting pro- and anti-inflammatory cytokines (16). In this review, we will provide a brief overview of B-cell development and differentiation, then discuss the mechanistic pathways of B-cell activation and differentiation, followed by a review of the transcriptional regulation of germinal center (GC) B cells, PCs, and memory B cells as they relate to solid organ transplant rejection.

## Overview of B-cell development and differentiation

B-cell development starts as hematopoietic stem cells in the fetal liver at birth and continues in the bone marrow where stromal cells provide cytokines and chemokines to stimulate hematopoiesis (17). Known as “cellular niches,” these microenvironments control B-lymphocytic behavior within the bone marrow during development. The earliest precursors, pre–pro-B cells, require CXC chemokine ligand (CXCL) 12 produced by a small population of stromal cells (18). These stromal cells are scattered throughout the bone marrow and away from interleukin (IL)-7-secreting cells, which cause the maturation of B-cell precursors.

Prior to B-cell lymphopoiesis, hematopoietic stem cells differentiate into common lymphoid progenitor cells that express factors such as c-kit and IL-7Rα (19). Activation of these receptors causes expression of transcription factors E2A and early B-cell factor, which cause these progenitor cells to develop into pro-B cells (20). Pro-B cells in the bone marrow undergo V(D)J recombination, resulting in IgM-expressing immature B cells (21), which migrate to the spleen. These early B cells then become immunoglobulin (Ig)D- and IgM-expressing mature B cells that are ready to be activated by foreign antigens (22).

Two signals are required for these mature B cells to differentiate into antibody-secreting PCs. The first signal is from antigen-coupled B-cell receptors and the second signal is from T-cell or non-T-cell-related antigens. T-cell-independent antigens, such as lipopolysaccharides and glycolipids, cause the differentiation of B cells into short-lived PCs that produce low-affinity antibodies (23). T-cell-dependent activation, *via* antigen stimulation and follicular helper T-cell (Tfh) interaction, causes differentiation into short-lived PCs or cell entry into germinal centers (GCs) (24). It is in GCs where B cells evolve into high-affinity B-cell receptors (BCRs) *via* mutation and selection by CD4^+^ T cells (25). Here, B cells also develop memory in the form of long-lived PCs and memory B cells (26).

There are two important modulators of B-cell survival: B-cell-activating factors of the tumor necrosis factor (TNF) family (gene name *TNFSF13b*) (BAFF) and TNF ligand superfamily member 13 (APRIL) (27). Both ligands have three receptors, namely, TNF receptor superfamily member 13C [also known as BAFF receptor (BAFF-R) or BLyS receptor 3 (BR3)], TNF receptor superfamily member 17 [also known as B-cell maturation antigen (BCMA)], and TNF receptor superfamily member 13B [also known as transmembrane activator and cyclophilin ligand interactor (TACI)]. BAFF, also known as B-lymphocyte stimulator (BLyS) and TNF-APOL-related leukocyte-expressed ligand (TALL-1), activates nuclear factor kappa B (NF-κB) signaling pathways upon interaction with BAFF-R. This trigger is important for B-cell survival as it signals the formation and maintenance of B cells (28). In the mouse model, overproduction of BAFF leads to autoimmune diseases like systemic lupus erythematosus (SLE) in humans (29), whereas gene deletion of BAFF prevents the development of an SLE-type disease (30). BAFF and APRIL are produced by myeloid cells, lymphoid cells, and toll-like receptor 9 (TLR9)-activated plasmacytoid dendritic cells and IL-2-activated natural killer cells (31). APRIL has been identified as being important in antibody class switching and plasma cell survival (32). Unlike BAFF, overproduction of APRIL in the animal model does not result in an SLE-type disease (33), but may be a target of interest for inhibiting SLE development in a mouse model (34).

The identification of these factors has led to the FDA-approved monoclonal antibody (mAb) belimumab, specific for BAFF (35), used to halt the immune response in SLE. Although belimumab has shown to have efficacy in reducing SLE disease (35, 36), belimumab has not shown the same results in kidney transplantation. In a double-blind, randomized, placebo-controlled phase 2 trial, Banham et al. observed that treatment with belimumab did not significantly reduce the number of naive B cells in 24 weeks (37). In the United States, studies are underway to further evaluate these findings.

## Transcriptional regulation of germinal center B-cell formation

**Figure 1:**
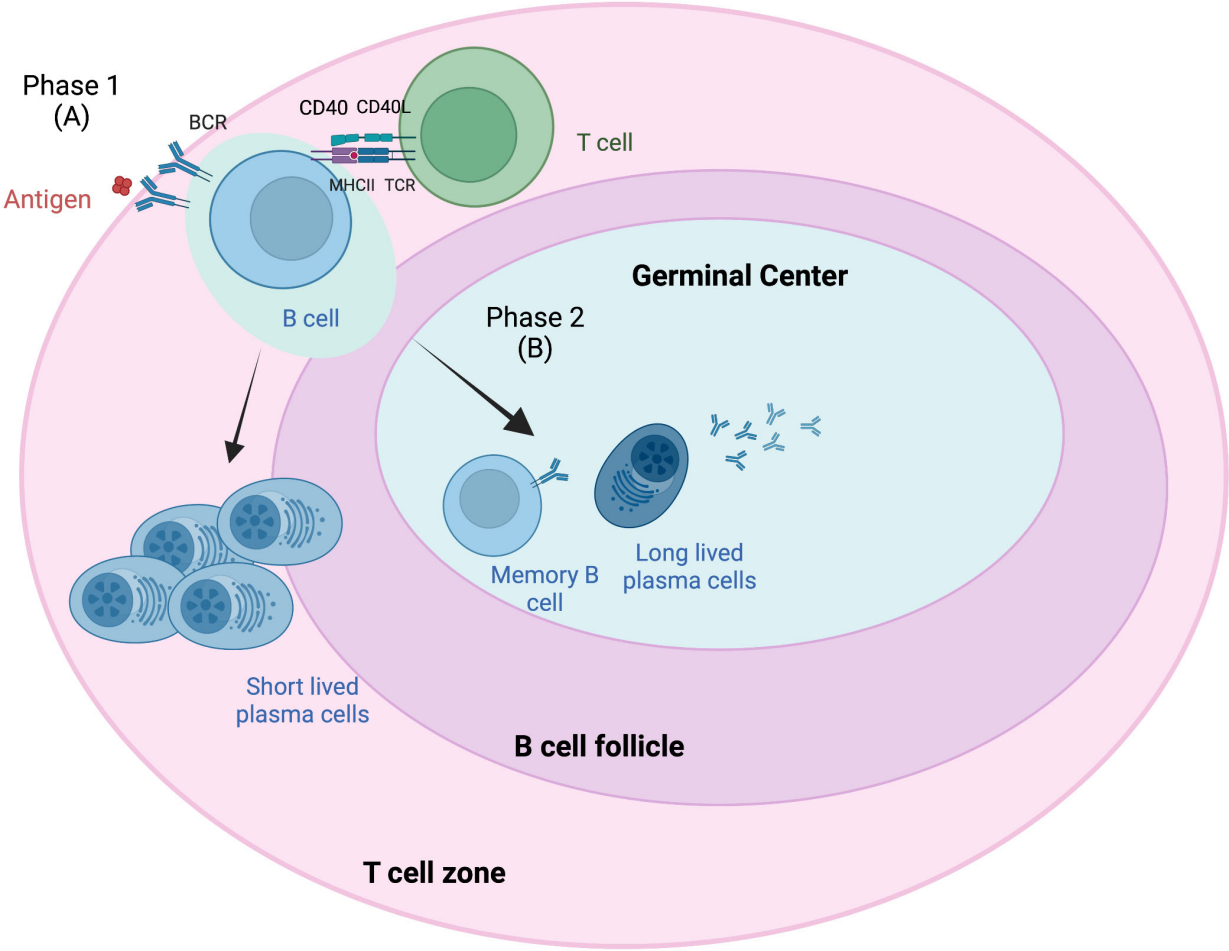
Germinal center response overview. The germinal center (GC) response consists of two phases (39). Phase 1 **(A)** involves the presentation of B-cell receptor (BCR)-bound antigens on major histocompatibility complex (MHC) class II T cells following antigen attachment and presentation to BCR on naive B cells. These B cells migrate to the interfollicular border of the T- and B-cell zones, causing cell activation, proliferation, and long-term interactions with antigen-specific T cells. Some of these B cells become short-lived plasma cells (PCs). Most of these activated B cells enter phase 2 **(B)**, where they differentiate into long-lived PCs and memory B cells in the GC. B-cell access into the GC has been attributed to interclonal competition for T-cell-associated signals, which occurs outside the follicles and prior to GC formation. Adapted from Verstegen et al. (40) Figure created with BioRender.com.

GCs are dynamic structures within peripheral lymphoid organs where T-cell-dependent B-cell selection occurs (38). Antigen-driven PCs and memory B cells from naive B cells occur in secondary lymphoid tissue in B-cell follicles and in GCs in two phases (**Figure 1**) (39). Phase 1 starts when antigens attach to the BCR on naive B cells, which then process and present BCR-bound antigen on major histocompatibility complex (MHC) class II molecules (41). This results in short-lived PCs and GC B cells that form in B-cell follicles. These B cells migrate to the interfollicular border of the T- and B-cell zones, causing cell activation, proliferation, and long-term interactions with antigen-specific T cells (42). Most of these activated B cells enter phase 2, in which they differentiate into long-lived PCs and memory B cells in the GC. This B-cell access into the GC has been attributed to interclonal competition for T-cell-associated signals, which occurs outside the follicles (43) and prior to GC formation.

**Figure 2:**
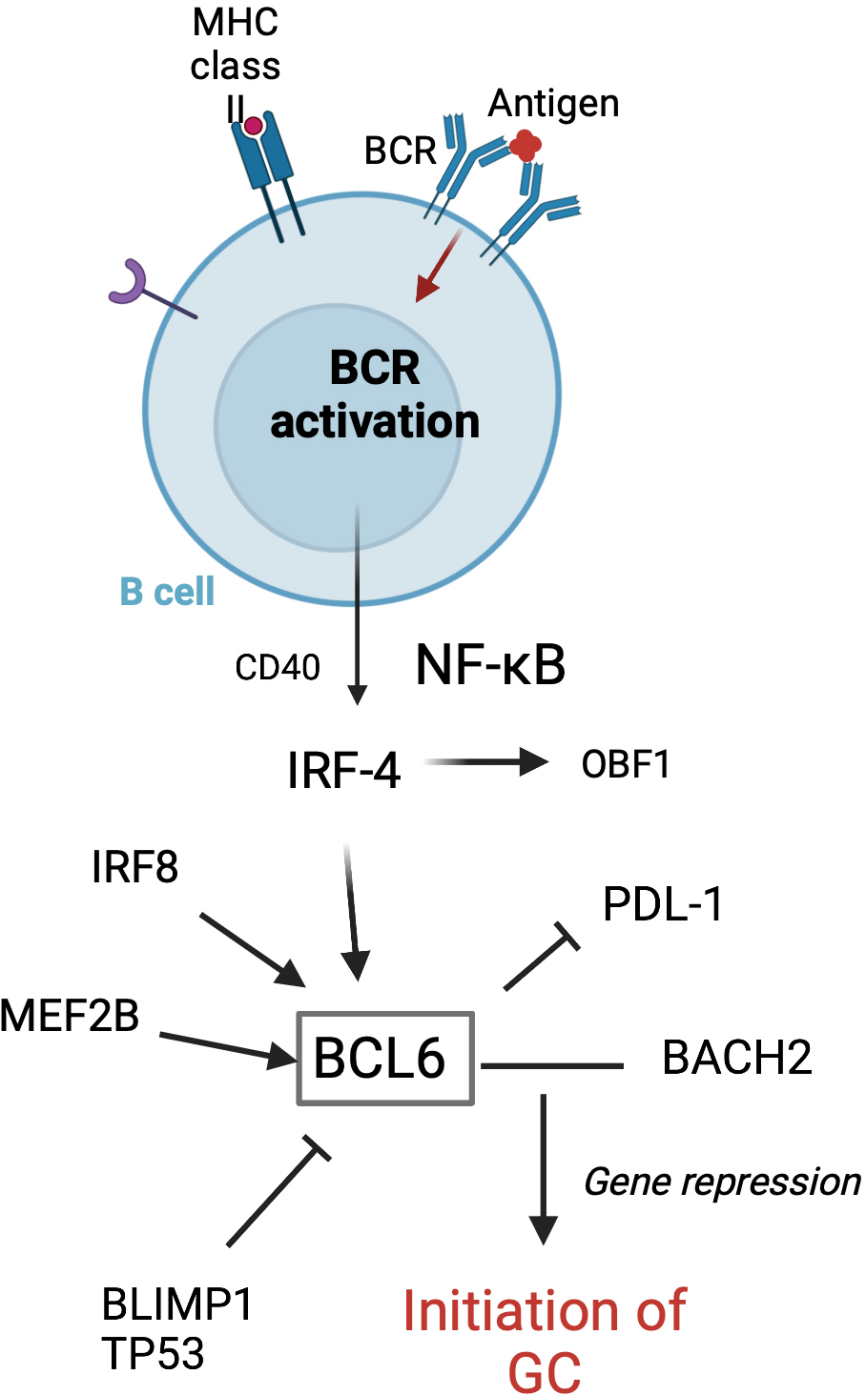
Transcription factors involved in the initiation of GC formation in follicular B cells. GC formation starts with BCR activation following antigen attachment. BCL-6 is essential for the initiation of the GC. MEF2B, IRF8, IRF4, BLIMP1, and TP53 are involved in regulating the BCL-6 expression. BCL-6 and BACH2 work to repress gene expression, thus allowing the GC program to proceed (45). Adapted from Song et al. (46) Figure created with BioRender.com.

Tfh cells play a crucial role in GC formation and the regulation of GC B cells. Tfh cells are a distinct subset of antigen-activated CD4^+^ T cells that express chemokine receptor (CXCR) 5 and B-cell lymphoma 6 (BCL-6). CXCR5 is a central marker of Tfh cells and shown to be required by B cells for entry into follicles (44). BCL-6 is a transcriptional repressor and master regulator of Tfh cells and GC B cells (**Figure 2**). This key transcription factor is required for Tfh cell differentiation and is a potent antagonist of B-lymphocyte-induced maturation protein 1 (BLIMP1) (47). Specifically, BCL-6 works to silence PD-1 ligands in GC B cells by binding the promoter region of PD-L1 and intron of PD-L2 (48). GC precursor cells deficient in BCL-6 cannot migrate to the center of the follicle and become high-affinity, class-switched memory B cells and long-lived PCs (49). Additionally, a recent study in mice by Robinson et al. has suggested that the amount of BCL-6 in B cells shortly after antigen activation determines B-cell fate into and toward the GC (50).

It was commonly believed that the GC is also where immunoglobulin (Ig) class-switch recombination (CSR) occurs. CSR is an intrachromosomal DNA rearrangement of the Ig heavy chain locus, thus allowing mature B cells to express antibodies of IgA, IgG, or IgE classes without altering the specificity for the antigen. CSR relies on the activation of activation-induced cytidine deaminase (51), uracil-DNa glycosylase (UNG), and apurinic-apyrimidinic endonuclease 1 (APE1) to target switch (S) regions (52). Activation-induced cytidine deaminase (AID) is essential for both somatic hypermutation (SHM) and CSR, and is expressed primarily in GCs (53). However, in a mouse model, Roco et al. showed that CSR is initiated over the first few days during the primary immune response (at the T-cell to B-cell interaction) and stops after B cells become GC cells and SHM starts (54). SHM is a major component of affinity maturation, in which the variable regions of the Ig gene produce high-affinity antibodies. The timeline of CSR suggests that CSR occurs outside the GC and restricting CSR in GC B cells potentially keeps a reservoir of more “naive-like” memory B cells. This is important as IgM^+^ memory B cells are considered more stable over time compared to that of class-switched memory B cells, and they can be rapidly activated in the setting of antigen interaction (infection or foreign pathogen) (55). These new discoveries may help us to understand the generation of pathogenic antibodies during allograft reaction (56).

Tfh cells exhibit seven distinction functions that affect B-cell activity: proliferation, survival, PC differentiation, SMH, CSR, adhesion, and attraction (44). Tfh cells express the CD40 ligand (CD40L) that provides a helper signal for maintaining B-cell survival, and produce IL-21 for promoting cell proliferation and differentiation. CD40L stimulation is a dominant mechanism for T-cell to B-cell interaction. T-cell-derived IL-4 and IL-21 cause transcriptional activation of signal transducer and activator of transcription (STAT) 6 and STAT4, which promote BCL-6 and the subsequent confinement of B cells in the GC (49).

The GC becomes divided into dark and light zones (DZ and LZ), which are organized by chemokines CXCR4 and CXCR5, respectively. CXCR4 expression, a response to CXCL12, helps B cells localize in the DZ. There, GC B cells undergo SMH, which generates a wide repertoire of antigen specificity (26). This occurs through AID, which induces DNA damage in the Ig genes that is then converted into mutations by DNA repair enzymes (51). BCL-6 must be co-expressed to repress the DNA damage response program that would otherwise induce apoptosis. Upregulation of BCL-6 appears to facilitate the migration of GC precursor B cells to the center of the follicle and enhance integrin-dependent conjugation of B and T cells (49). Forkhead box protein O1 (FOXO1) with BCL-6 represses the expression of BLIMP1 to maintain this GC DZ B-cell program (57). Positively selected GC B cells eventually acquire avian myelocytomatosis virus oncogene cellular homolog (c-Myc) expression, which allows LZ B cells to re-enter the DZ (58). However, recent *ex-vivo* experiments by Radtke et al. showed that subsets of DZ cells expressed BLIMP-1 even during low proliferation states (59). The authors speculated that BLIMP-1 may be induced in certain DZ cells even in the absence of Tfh cells. Similarly, Santamaria et al. observed that human GC B cells that do not express CD23 are likely to differentiate into PC (59). Pae et al. reported that certain DZ B cells appeared to adopt a distinct E2F^high^/c-Myc^low^ profile, allowing for a rapid and continuous cyclin D3-dependent proliferation despite the absence of signaling from Tfh cells (60). This is most evident in malignant processes, such as Burkitt’s lymphoma, where the gain of function mutation leads to increased DZ proliferation and subsequent B-cell lymphoproliferation.

The LZ is characterized by Tfh cells, follicular dendritic cells, and macrophages. There are fewer B cells here compared to the DZ. Dark zone B cells move to the LZ *via* CXCR5, where the Tfh cells, follicular dendritic cells, and macrophages help select modified B-cell receptors for antigen binding. Chemoattraction by CXCL13, a ligand for CXCR5 and GC Tfh cells, causes the recruitment of B cells to co-localize with Tfh cells. It can also modify B-cell adhesion and lymphotoxin synthesis, as CXCL13 has cytokine-type functions. Through positive selection, newly generated LZ B cells that do not produce favorable antibodies undergo apoptosis. B cells can recirculate between LZ and DZ to optimize antigen affinity through repeated SHM, which leads to the generation of high-affinity memory B cells and PCs. In GC B cells, signals from CD40, *via* NF-κB, and BCR are required to induce c-Myc (61). These two signals induce p-S6 in GC B cells, which allows for cell cycle entry. Using single-cell RNA sequencing, Nakagawa et al. identified three different subpopulations of c-Myc^+^, suggesting how positive selection can lead to clonal diversity during affinity maturation (62). Liu et al. observed in a mouse model that GC B cells upregulate C-C motif chemokine ligand 22 and 17 (CCL22 and CCL17, respectively), which interacts with CCR4 on Tfh cells (63). This highlights higher-affinity GC B cells, resulting in affinity maturation. B cells without CCL22 and CCL17 receive less T-cell help and are unable to undergo affinity maturation. Similarly, Li et al. showed that strong CD40 and BCR signals caused Cbl degradation resulting in increased interferon regulatory factor (IRF) 4 expression and exit from GC affinity selection (64).

In disease processes with dysregulated GC reactions, such as B-cell lymphoma and autoimmune diseases, B cells with mutated BCRs have also been shown to enter the LZ and compete for antigen expression on follicular dendritic cells (65, 66). Those B cells with the highest affinity for the antigen can outcompete other B cells and present peptides on class II MHC. The cells can then receive positive selection signals when they interact with Tfh. However, the ability for BCR mutations to undergo affinity maturation is likely specific to the genetic signature of the disease process. Not all cells with BCR mutations go to the LZ. A recent study by Stewart et al. suggests that mutated BCRs are replaced following SHM, as they rarely reach the LZ (67). These mutated cells undergo apoptosis, whereas functional BCRs re-enter the cell cycle or migrate to the LZ. Additionally, in the murine model, IL-21 has shown to be important in not only sustaining the GC but also promoting the entry of antigen-specific LZ B cells into the cell cycle (68). In IL-21-deficient mice, there was a deficit of LZ B cells entering the S phase of the cell cycle. In the presence of IL-21, Tfh cell interaction with LZ B cells showed a transient expression of c-*Myc*, which appears necessary for centroblast proliferation (68). Gene expression analysis has shown that deficiency in c-*Myc* expression by Tfh cells leads to deregulation of DZ B-cell division and subsequent B-cell lymphoma (69). There are also lower-affinity B cells that do not undergo apoptosis and remain in the GC (62). These retained cells have been associated with aberrations in Fas and BCL-2, which are also seen in B-cell lymphoma (70).

There are several key factors associated with GC development. BCL-6 is key for the initiation of the GC reaction, including the downregulation of sphingosine-1-phosphate receptor type 1 (S1PR1) which otherwise helps mediate B-cell trafficking out of the follicle (9). Myocyte-specific enhancer factor (MEF2C) is required for GC formation, as it has a role in B-cell proliferation immediately after antigen stimulation (71). Interferon regulatory factors (47) are a family of transcription factors that play important roles in innate and adaptive immune responses, such as immune cell development and differentiation and pathogen response (72). Interferon regulatory factor 4 (IRF4) regulates myeloid cell development, thus playing an important role in regulating the inflammatory response. IRF4 has been found to both activate and repress BCL-6 transcription (73) and regulate Tfh cells (74). Early in GC formation, a transient and low expression of IRF4 leads to BCL-6 expression (75), while a more sustained and high level of IRF4 with BLIMP1 upregulates X-box binding protein 1 (XBP1), a transcription factor required for PC differentiation (73). In IRF4 knockout models, IRF4^−^ B cells failed to differentiate into GC B cells (73). Additionally, BCL-6 has also been shown to increase complement activation required for positive selection in the murine model (76). As a transcriptional repressor, BCL-6 inhibits complement regulators thus allowing C3aR/C5aR signaling required for positive selection despite the presence of Tfh.

Overall, there are multiple distinct transcription factors that work in a complex but cohesive manner to activate or repress GC development. Understanding how GC development occurs is important in understanding how PC differentiation is regulated. This becomes especially pertinent when evaluating alloimmunity in the setting of chronic rejection in solid organ transplantation.

## Transcriptional regulation of plasma cell differentiation

**Figure 3:**
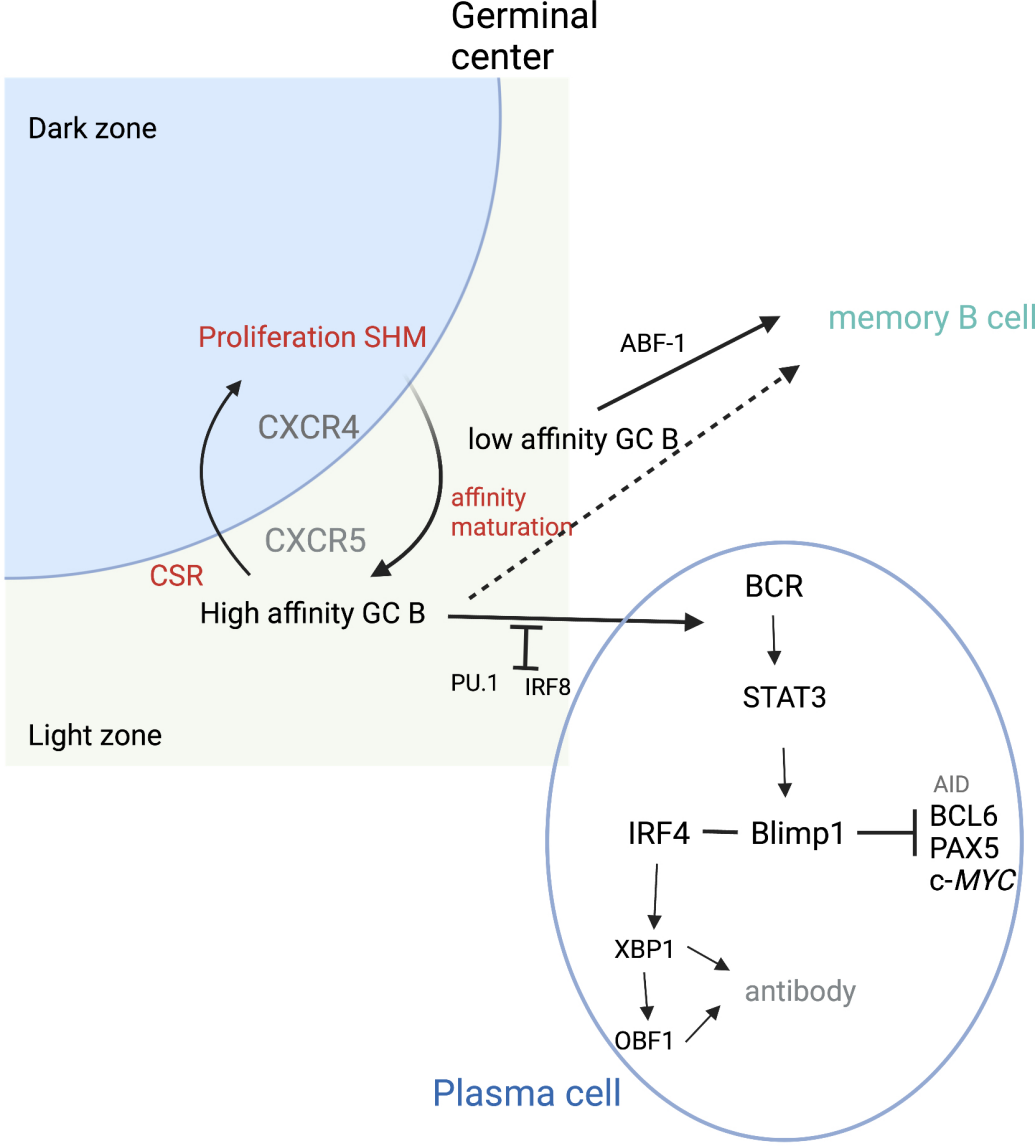
Plasma cell differentiation. During cycling between DZ and LZ, B cells undergo SMH in the DZ and then migrate to the LZ where affinity maturation occurs. High-affinity B cells can then differentiate into plasma cells. PU.1 and IRF8 negatively regulate plasma cell differentiation. IRF4 and BLIMP1 form the central axis in establishing plasma cells. BLIMP1 further suppresses the expression of AID, BCL6, PAX5, and c-*Myc* genes and finally terminates the GC program. BLIMP1 positively regulates Xbp1 expression, making the cells ready for antibody production and secretion. ABF-1 promotes memory B-cell differentiation, which can occur following both high- and low-affinity maturation. Adapted from Song et al. (46) Figure created with BioRender.com.

Antibody production occurs in terminally differentiated B cells or PCs. Following GC exit, B cells either become memory cells or PCs (**Figure 3**). PC fate has been associated with the degree of antigen affinity (77). As mentioned previously, NF-κB pathways are activated by CD40 signals, inducing c-*Myc* or IRF4 expression. High sustained levels of IRF4 expression are required for PC differentiation (73). BLIMP1, the transcription repressor that can induce plasmablast differentiation, can also increase the level of IRF4 (78). High levels of IRF4 downregulate BCL-6 and induce BLIMP1 expression. Together, IRF4 and BLIMP1 repress the GC program and activate the PC differentiation program. STAT3 regulates PC differentiation by promoting cell survival through activation of such prosurvival genes as B-cell leukemia 2-like 1 (BLC2L1) and myeloid cell leukemia-1 (MCL1) (79). Conversely, IRF8 with PU.1 transcription factor inhibits GC B-cell differentiation to PCs (80). The balance between IRF4 and IRF8 may be crucial in determining B-cell fate (80).

There are several cytokines that appear to play important roles in B-cell proliferation. IL-21 is the most potent cytokine for stimulating PC differentiation and is key in B-cell proliferation (81). BTB domain and CNC homolog 2 (BACH2) and paired box 5 (PAX5) are upregulated in the light zone GC B cells. BACH2 appears to regulate light zone B cells to commit to the memory B-cell pathway by repression of BCL-6 (45), as BACH2 deficiency leads to lower memory B-cell differentiation (82). Activated B-cell factor 1 (ABF-1) blocks the PC differentiation program (83). In the ABF-1-ER mouse model, ABF-1 promotes GC formation and memory B-cell differentiation. BCL-6 inhibits PC differentiation by repressing PR/SET domain 1 (*PRDM1*) expression (84). STAT5 appears to modulate BCL-6 expression, which then represses *PRDM1* expression, thus promoting memory B-cell differentiation. When *PRDM1* expression occurs, BLIMP1 is encoded, thus allowing for PC differentiation.

PC differentiation is also controlled by epigenetic programs. In an *in-vivo* T-cell-independent B-cell differentiation model, Scharer et al. described a cellular division-dependent *cis*-regulatory element (85). Enhancer of zeste 2 polycomb repressive complex 2 (EZH2), a catalytic subunit of the polycomb complex, provides a repressive histone modification of H3K27m3 (trimethylation of histone H3 lysine 27) necessary for B-cell development during the GC reaction (86). Chemical inhibition of EZH2 resulted in enhanced PC formation *ex vivo* (85), as well as premature expression of BLIMP1. Barwick et al. showed that *de-novo* DNA methylation limits the regulation of PC differentiation by repressing the PC chromatin state (87).

PC generated during the GC reaction migrates to the bone marrow, where PCs can stay quiescent for a long duration of time. This may provide an important clue as to the chronic activation of the immune response during solid organ transplantation, despite immunosuppressive therapy.

## Allogenic B-cell response and transplant rejection

Although alloreactive T cells are traditionally considered to be the primary culprit in mediating allograft reaction, it is the T- and B-cell interaction that plays a key role in generating humoral alloimmune responses so detrimental to the longevity of the solid organ transplant. As discussed previously, memory B cells are generated from low-affinity GC B cells in the light zone and will eventually enter the circulation as patrolling cells or stay in lymphoid or target organs. Solid organ allografts, such as in kidney transplantation, become ectopic lymphoid-like structures (88), where alloreactive B cells can form an extrafollicular response as short-lived PCs or return to B-cell follicles to initiate a GC reaction (89). In the GC, B cells can undergo SHM and compete for Tfh cell interactions for high antigen affinity. These B cells can then transition into long-lived PCs, while the lower affinity B cells remain as a more diverse pool of B cells (90). The lower affinity B cells can then circulate in their quiescent form until a recognizable antigen is encountered.

Follicular regulatory T (Tfr) cells are a unique subset of forkhead box P3 (FoxP3)^+^ regulatory T cells (Tregs) that inhibit Tfh and B cells. Tfr cells downregulate the co-stimulatory molecule cluster of differentiation 86 (91) (CD86) on B cells, producing inhibitory cytokines such as IL-10 (92). Like other Tregs, Tfr cells can also express Helios, a marker reflecting enhanced immunosuppressive function (93). The balance between Tfr and Tfh is crucial for immune homeostasis and tolerance, as an aberrant Tfr/Tfh ratio has been linked to autoimmune diseases (94) such as systemic lupus erythematosus and myasthenia gravis (95). In kidney transplantation, antibody-mediated rejection has also been associated with an imbalance in circulating Tfr and Tfh cells (96).

As described previously, Tfh cells play an important role in alloreactivity and provide help to alloantigen-activated B cells. This is also evident in solid organ transplantation. Tfh cells are present in kidney transplant allografts with the pathological findings of T-cell-mediated rejection (97, 98). They can also stimulate alloantibody production, which mediates humoral responses against the transplanted organ (99). Tfr cells are found to be impaired in this setting, leading to an uncontrolled allogeneic response from Tfh cells following transplantation. Similarly, Louis et al. observed a loss of regulatory T and B cells in kidney transplant recipient serum in the setting of ABMR (100). Similar to Treg cells, transitional B cells (Trb) play a role in immune suppression, as the authors found that Trb suppressed Tfh activation, Tfh- to B-cell activation, and antibody generation *in vivo*.

Immunosuppression has been found to alter the absolute number of Tfr and Tfh cells in kidney transplant recipients. Niu et al. showed that peripheral Tfr cells significantly decreased with calcineurin inhibitor-based therapy (101), while Tfh did not change. When transplant recipients were treated with alemtuzumab, a monoclonal antibody that binds CD52, there were lower numbers of total and subset Tfr cells and total Tfh cells, suggesting an impairment of the homeostatic proliferation of Tfr cells. Similarly, Macdeo et al. showed a significant decrease in Tregs in peripheral blood mononuclear cells (PBMCs) of kidney transplant recipients compared to that of non-immunosuppressed healthy controls (102). Despite lymphodepletion, this shift to Tfh cells may result in increased memory B-cell formation and PCs, potentially increasing the risk of antibody-mediated rejection. Clinically, patients treated with alemtuzumab have been shown to have a higher incidence of chronic antibody-mediated rejection and donor-specific antibodies despite this antirejection therapy (103).

Another therapy being used on kidney transplant recipients is belatacept, a cytotoxic T-lymphocyte–associated antigen 4 (CTLA-4) co-stimulatory inhibitor. Belatacept targets CD80/86 to prevent interaction with CD28 and CTLA-4, thus preventing the T- and B-cell interactions required for class-switch recombination and GC response. In early clinical trials, belatacept demonstrated a significant reduction in the incidence of DSA (104) but increased early rejection in this group (105, 106), suggesting the need for combination treatment. In non-human primate data, Schroder et al. showed feasibility of using co-stimulatory blockade *via* CTLA-4 Ig with plasma cell inhibition (*via* carfilzomib) for pretransplant desensitization and ABMR treatment (107, 108). The authors report that the selective targeting of CD28 reduced DSA, Tfh, and B-cell expansion while preserving Treg cells and promoting naive T and B cells. With strong evidence supporting the reduction of DSA using belatacept alone and with combination therapy, there are two ongoing clinical trials investigating the use of plasma cell inhibitors (carfilzomib (109) or daratumumab (110)) and belatacept for desensitization in patients awaiting kidney transplant.

Humoral immunity against the allograft may be due to a steady production of donor-specific antibodies created from alloreactive T- and B-cell interactions, despite immunosuppressive therapy. In mouse studies, B cells recognizing one specific donor MHC can receive stimulatory help from T cells specific to another donor MHC, thus stimulating alloantibodies against an allograft (111). When naive CD4^+^ T cells were depleted by co-stimulation blockade of CD40/CD154, interferon-gamma (IFN-γ)-producing memory CD4^+^ T cells were still able to activate B cells (112). Thus, alloantibody response can be initiated prior to transplant due to the diverse repertoire in memory B cells (113). This may be undetectable in the serum, as donor-reactive memory B cells are not measured during immunologic risk assessments prior to transplant. Additionally, the failure of immunosuppressive therapy suggests that there is a heterogeneity of B-cell lymphocyte populations and functions.

Targeting memory B-cell longevity may be the answer to resolving immunosuppression failure in solid organ transplantation. In vaccine studies, B-cell-intrinsic autophagy has been shown to be critical for memory B-cell longevity (114) and maintenance (115). Chen et al. observed that autophagy was found to be essential for memory B-cell maintenance against viral infections in mice. The authors found decreased spontaneous cell death and increased B-cell survival in the presence of autophagy, as evidenced by increased FOXO1 and FOXO3 expression in memory B cells over time (115). In the absence of autophagy, there was increased oxidative stress and memory B-cell loss. FOXO3 silencing also led to a suppression of autophagy gene expression in memory B cells. In a subsequent study by Kodali et al., the authors demonstrated that in the presence of Nix- and Bnip3-mediated mitochondrial autophagy (116), there was maintenance of metabolic quiescence and longevity of memory B cells. In a transplant mouse model, Fribourg et al. showed the effect of autophagy in memory B cells in transplantation. By inhibiting autophagy through the chemical inhibitor 3-methyladenine, the authors demonstrated a decrease in DSA responses to alloantigens in treated versus control mice. Such important findings provide potential therapeutic targets against autophagy, which may help mitigate the B-cell response in clinical transplantation.

## Summary

The intrinsic relationship between T- and B-cell pathways results in a complex interplay activating rejection pathways in solid organ transplantation. Transcriptional factors, such as IRF4, initially appear to affect allogenic T-cell effector pathways (117), modulating the inflammatory response. Within this response, B-cell pathways become activated (118), creating a chronic pathway of rejection that is currently untreatable with current immunosuppressive therapies. Emerging evidence suggests that transcriptional regulation through B cells is the key to transplant tolerance. Elucidating various B-cell subtypes, like T-cell subtypes, may also be necessary to better understand early B-cell response on a transcriptional level. Future modalities investigating both metabolic and epigenetic regulation will prove useful in understanding the complexities within B-cell response in transplantation. Such information can provide important insights as to the early detection of immune risk prior to transplantation, specifically as it relates to B-cell response, as well as the development of therapeutic modalities for both prophylaxis and treatment against chronic allograft rejection.

## Author contributions

SY and WC conceived and wrote the review. SY, AG, and WC contributed to the article and approved the submitted version.

## Funding

SY and WC were internally funded. SY was also funded by the Houston Methodist Hospital Clinical Scholars award.

## Conflict of interest

The authors declare that the research was conducted in the absence of any commercial or financial relationships that could be construed as a potential conflict of interest.

## Publisher’s note

All claims expressed in this article are solely those of the authors and do not necessarily represent those of their affiliated organizations, or those of the publisher, the editors and the reviewers. Any product that may be evaluated in this article, or claim that may be made by its manufacturer, is not guaranteed or endorsed by the publisher.

## Glossary

**Table:**

ABF-1	activated B-cell factor 1
AID	activation-induced cytidine deaminase
ABMR	antibody-medicated rejection
APE1	apurinic-apyrimidinic endonuclease 1
c-Myc	avian myelocytomatosis virus oncogene cellular homolog
BAFF	B-cell activation factor of the TNF family
BCL-6	B-cell lymphoma 6
BCL2LC1	B-cell leukemia 2-like 1
BCMA	B-cell maturation antigen
BCR	B-cell receptor
BCRP	B-cell receptor protein
BLIMP1	B-lymphocyte-induced maturation protein 1
BR3	BLyS receptor 3
BACH2	BTB domain and CNC homolog 2
CCL22	C-C motif chemokine ligand 22
CCL17	C-C motif chemokine ligand 17
CD40L	CD40 ligand
CXCR	chemokine receptor
CD	cluster of differentiation
CXCL	CXC chemokine ligand
CTLA-4	cytotoxic T-lymphocyte&ndash;associated antigen 4
DZ	dark zone
DSAs	donor-specific antibodies
EZH2	enhancer of zeste 2 polycomb repressive complex 2
Tfh	follicular helper T cells
Tfr	follicular regulatory T cells
FOXO1	forkhead box protein O1
FOXO3	forkhead box protein O3
FoxP3	forkhead box P3
GC	germinal center
HLA	human leukocyte antigen
Ig	immunoglobulin
IFN-&gamma;	interferon-gamma
IRF	interferon regulatory factor
IL	interleukin
LZ	light zone
MHC	major histocompatibility complex
mAb	monoclonal antibody
MCL-1	myeloid cell leukemia-1
MEF2C	myocyte-specific enhancer factor
NK	natural killer
NF-&kappa;B	nuclear factor kappa B
PAX5	paired box 5
PBMCs	peripheral blood mononuclear cells
PCs	plasma cells
PRDM1	PR/SET domain 1
STAT	signal transducer and activator of transcription
SHM	somatic hypermutation
S1PR1	sphingosine-1-phosphate receptor type
SLE	systemic lupus erythematosus
TALL-1	TNF-APOL-related leukocyte expressed ligand
TCMR	T-cell-mediated rejection
TACI	transmembrane activator and cyclophilin ligand interactor
Trb	transitional B cells
H3K27m3	trimethylation of histone H3 lysine 27
TNF	tumor necrosis factor
UNG	uracil-DNa glycosylase
XBP1	X-box binding protein

## References

[1] Hart A Lentine KL Smith JM Miller JM Skeans MA Prentice M. OPTN/SRTR 2019 annual data report: Kidney. Am J Transplant (2021) 21(S2):21–137. doi: 10.1111/ajt.16502 33595191

[2] Poggio ED Augustine JJ Arrigain S Brennan DC Schold JD. Long-term kidney transplant graft survival–making progress when most needed. Am J Transplant (2021) 21(8):2824–32. doi: 10.1111/ajt.16463 33346917

[3] Wekerle T Segev D Lechler R Oberbauer R. Strategies for long-term preservation of kidney graft function. Lancet (2017) 389(10084):2152–62. doi: 10.1016/S0140-6736(17)31283-7 28561006

[4] Böhmig GA Eskandary F Doberer K Halloran PF. The therapeutic challenge of late antibody-mediated kidney allograft rejection. Transpl Int (2019) 32(8):775–88. doi: 10.1111/tri.13436 PMC6850109 30955215

[5] Eskandary F Jilma B Mühlbacher J Wahrmann M Regele H Kozakowski N. Anti-C1s monoclonal antibody BIVV009 in late antibody-mediated kidney allograft rejection-results from a first-in-patient phase 1 trial. Am J Transplant (2018) 18(4):916–26. doi: 10.1111/ajt.14528 28980446

[6] Moreso F Crespo M Ruiz JC Torres A Gutierrez-Dalmau A Osuna A. Treatment of chronic antibody mediated rejection with intravenous immunoglobulins and rituximab: A multicenter, prospective, randomized, double-blind clinical trial. Am J Transplant (2018) 18(4):927–35. doi: 10.1111/ajt.14520 28949089

[7] Eskandary F Dürr M Budde K Doberer K Reindl-Schwaighofer R Waiser J. Clazakizumab in late antibody-mediated rejection: study protocol of a randomized controlled pilot trial. Trials (2019) 20(1):37. doi: 10.1186/s13063-018-3158-6 30635033 PMC6329051

[8] Gago M Cornell LD Kremers WK Stegall MD Cosio FG. Kidney allograft inflammation and fibrosis, causes and consequences. Am J Transplant (2012) 12(5):1199–207. doi: 10.1111/j.1600-6143.2011.03911.x 22221836

[9] Huang C Gonzalez DG Cote CM Jiang Y Hatzi K Teater M. The BCL6 RD2 domain governs commitment of activated b cells to form germinal centers. Cell Rep (2014) 8(5):1497–508. doi: 10.1016/j.celrep.2014.07.059 PMC4163070 25176650

[10] Jindra PT Jin YP Rozengurt E Reed EF. HLA class I antibody-mediated endothelial cell proliferation *via* the mTOR pathway. J Immunol (2008) 180(4):2357–66. doi: 10.4049/jimmunol.180.4.2357 18250445

[11] Valenzuela NM McNamara JT Reed EF. Antibody-mediated graft injury: complement-dependent and complement-independent mechanisms. Curr Opin Organ Transplant (2014) 19(1):33–40. doi: 10.1097/MOT.0000000000000040 24316758 PMC4080796

[12] Bentall A Tyan DB Sequeira F Everly MJ Gandhi MJ Cornell LD. Antibody-mediated rejection despite inhibition of terminal complement. Transpl Int (2014) 27(12):1235–43. doi: 10.1111/tri.12396 24990476

[13] Burbach M Suberbielle C Brochériou I Ridel C Mesnard L Dahan K. Report of the inefficacy of eculizumab in two cases of severe antibody-mediated rejection of renal grafts. Transplantation (2014) 98(10):1056–9. doi: 10.1097/TP.0000000000000184 24839895

[14] Valenzuela NM Reed EF. Antibodies in transplantation: the effects of HLA and non-HLA antibody binding and mechanisms of injury. Methods Mol Biol (2013) 1034:41–70. doi: 10.1007/978-1-62703-493-7_2 23775730 PMC3879955

[15] Philogene MC Jackson AM. Non-HLA antibodies in transplantation: when do they matter? Curr Opin Organ Transplant (2016) 21(4):427–32. doi: 10.1097/MOT.0000000000000335 27258575

[16] Karahan GE Claas FH Heidt S. B cell immunity in solid organ transplantation. Front Immunol (2016) 7:686. doi: 10.3389/fimmu.2016.00686 28119695 PMC5222792

[17] Tokoyoda K Egawa T Sugiyama T Choi BI Nagasawa T. Cellular niches controlling b lymphocyte behavior within bone marrow during development. Immunity (2004) 20(6):707–18. doi: 10.1016/j.immuni.2004.05.001 15189736

[18] Nagasawa T Hirota S Tachibana K Takakura N Nishikawa Kitamura S-i Y. Defects of b-cell lymphopoiesis and bone-marrow myelopoiesis in mice lacking the CXC chemokine PBSF/SDF-1. Nature (1996) 382(6592):635–8. doi: 10.1038/382635a0 8757135

[19] Barata JT Durum SK Seddon B. Flip the coin: IL-7 and IL-7R in health and disease. Nat Immunol (2019) 20(12):1584–93. doi: 10.1038/s41590-019-0479-x 31745336

[20] O'Riordan M Grosschedl R. Coordinate regulation of b cell differentiation by the transcription factors EBF and E2A. Immunity (1999) 11(1):21–31. doi: 10.1016/S1074-7613(00)80078-3 10435576

[21] Krangel MS. Gene segment selection in V(D)J recombination: accessibility and beyond. Nat Immunol (2003) 4(7):624–30. doi: 10.1038/ni0703-624 12830137

[22] Chung JB Silverman M Monroe JG. Transitional b cells: step by step towards immune competence. Trends Immunol (2003) 24(6):343–9. doi: 10.1016/S1471-4906(03)00119-4 12810111

[23] Hoffman W Lakkis FG Chalasani G. B cells, antibodies, and more. Clin J Am Soc Nephrol (2016) 11(1):137–54. doi: 10.2215/CJN.09430915 PMC4702236 26700440

[24] Nutt SL Hodgkin PD Tarlinton DM Corcoran LM. The generation of antibody-secreting plasma cells. Nat Rev Immunol (2015) 15(3):160–71. doi: 10.1038/nri3795 25698678

[25] Kurosaki T Kometani K Ise W. Memory b cells. Nat Rev Immunol (2015) 15(3):149–59. doi: 10.1038/nri3802 25677494

[26] Mesin L Ersching J Victora GD. Germinal center b cell dynamics. Immunity (2016) 45(3):471–82. doi: 10.1016/j.immuni.2016.09.001 PMC5123673 27653600

[27] Vincent FB Morand EF Schneider P Mackay F. The BAFF/APRIL system in SLE pathogenesis. Nat Rev Rheumatol (2014) 10(6):365–73. doi: 10.1038/nrrheum.2014.33 24614588

[28] Kreuzaler M Rauch M Salzer U Birmelin J Rizzi M Grimbacher B. Soluble BAFF levels inversely correlate with peripheral b cell numbers and the expression of BAFF receptors. J Immunol (2012) 188(1):497–503. doi: 10.4049/jimmunol.1102321 22124120

[29] Mackay F Woodcock SA Lawton P Ambrose C Baetscher M Schneider P. Mice transgenic for BAFF develop lymphocytic disorders along with autoimmune manifestations. J Exp Med (1999) 190(11):1697–710. doi: 10.1084/jem.190.11.1697 PMC2195729 10587360

[30] Jacob CO Pricop L Putterman C Koss MN Liu Y Kollaros M. Paucity of clinical disease despite serological autoimmunity and kidney pathology in lupus-prone new Zealand mixed 2328 mice deficient in BAFF. J Immunol (2006) 177(4):2671–80. doi: 10.4049/jimmunol.177.4.2671 PMC2896675 16888029

[31] Vincent FB Morand EF Mackay F. BAFF and innate immunity: new therapeutic targets for systemic lupus erythematosus. Immunol Cell Biol (2012) 90(3):293–303. doi: 10.1038/icb.2011.111 22231653

[32] Vincent FB Saulep-Easton D Figgett WA Fairfax KA Mackay F. The BAFF/APRIL system: emerging functions beyond b cell biology and autoimmunity. Cytokine Growth Factor Rev (2013) 24(3):203–15. doi: 10.1016/j.cytogfr.2013.04.003 PMC7108297 23684423

[33] Mackay F Schneider P. Cracking the BAFF code. Nat Rev Immunol (2009) 9(7):491–502. doi: 10.1038/nri2572 19521398

[34] Huard B Tran NL Benkhoucha M Manzin-Lorenzi C Santiago-Raber ML. Selective APRIL blockade delays systemic lupus erythematosus in mouse. PloS One (2012) 7(2):e31837. doi: 10.1371/journal.pone.0031837 22355399 PMC3280206

[35] Navarra SV Guzmán RM Gallacher AE Hall S Levy RA Jimenez RE. Efficacy and safety of belimumab in patients with active systemic lupus erythematosus: a randomised, placebo-controlled, phase 3 trial. Lancet (2011) 377(9767):721–31. doi: 10.1016/S0140-6736(10)61354-2 21296403

[36] Furie R Petri M Zamani O A phase III. Randomized, placebo-controlled study of belimumab, a monoclonal antibody that inhibits b lymphocyte stimulator, in patients with systemic lupus erythematosus. Arthritis Rheumatism. (2011) 63(12):3918–30. doi: 10.1002/art.30613 PMC5007058 22127708

[37] Banham GD Flint SM Torpey N Lyons PA Shanahan DN Gibson A. Belimumab in kidney transplantation: an experimental medicine, randomised, placebo-controlled phase 2 trial. Lancet (2018) 391(10140):2619–30. doi: 10.1016/S0140-6736(18)30984-X PMC7617033 29910042

[38] Klein U Dalla-Favera R. Germinal centres: role in b-cell physiology and malignancy. Nat Rev Immunol (2008) 8(1):22–33. doi: 10.1038/nri2217 18097447

[39] Avalos A Ploegh H. Early BCR events and antigen capture, processing, and loading on MHC class II on b cells. Front Immunol (2014) 5. doi: 10.3389/fimmu.2014.00092 PMC3948085 24653721

[40] Verstegen NJM Ubels V Westerhoff HV van Ham SM Barberis M. System-level scenarios for the elucidation of T cell-mediated germinal center b cell differentiation. Front Immunol (2021) 12. doi: 10.3389/fimmu.2021.734282 PMC8488341 34616402

[41] Hernández-Pérez S Vainio M Kuokkanen E Šuštar V Petrov P Forstén S. B cells rapidly target antigen and surface-derived MHCII into peripheral degradative compartments. J Cell Science (2019) 133(5):jcs235192. doi: 10.1101/775882 31780582

[42] Okada T Miller MJ Parker I Krummel MF Neighbors M Hartley SB. Antigen-engaged b cells undergo chemotaxis toward the T zone and form motile conjugates with helper T cells. PloS Biol (2005) 3(6):e150. doi: 10.1371/journal.pbio.0030150 15857154 PMC1088276

[43] Schwickert TA Victora GD Fooksman DR Kamphorst AO Mugnier MR Gitlin AD. A dynamic T cell-limited checkpoint regulates affinity-dependent b cell entry into the germinal center. J Exp Med (2011) 208(6):1243–52. doi: 10.1084/jem.20102477 PMC3173244 21576382

[44] De Silva NS Klein U. Dynamics of b cells in germinal centres. Nat Rev Immunol (2015) 15(3):137–48. doi: 10.1038/nri3804 PMC4399774 25656706

[45] Lahmann A Kuhrau J Fuhrmann F. Bach2 controls T follicular helper cells by direct repression of bcl-6. J Immunol (2019) 202(8):2229–39. doi: 10.4049/jimmunol.1801400 30833348

[46] Song S Matthias PD. The transcriptional regulation of germinal center formation. Front Immunol (2018) 9. doi: 10.3389/fimmu.2018.02026 PMC6134015 30233601

[47] Nutt SL Fairfax KA Kallies A. BLIMP1 guides the fate of effector b and T cells. Nat Rev Immunol (2007) 7(12):923–7. doi: 10.1038/nri2204 17965637

[48] Peng C Hu Q Yang F Zhang H Li F Huang C. BCL6-mediated silencing of PD-1 ligands in germinal center b cells maintains follicular T cell population. J Immunol (2019) 202(3):704–13. doi: 10.4049/jimmunol.1800876 30567732

[49] Kitano M Moriyama S Ando Y Hikida M Mori Y Kurosaki T. Bcl6 protein expression shapes pre-germinal center b cell dynamics and follicular helper T cell heterogeneity. Immunity (2011) 34(6):961–72. doi: 10.1016/j.immuni.2011.03.025 21636294

[50] Robinson MJ Ding Z Pitt C Brodie EJ Quast I Tarlinton DM. The amount of BCL6 in b cells shortly after antigen engagement determines their representation in subsequent germinal centers. Cell Rep (2020) 30(5):1530–1541.e1534. doi: 10.1016/j.celrep.2020.01.009 32023467

[51] Laidlaw BJ Cyster JG. Transcriptional regulation of memory b cell differentiation. Nat Rev Immunol (2021) 21(4):209–20. doi: 10.1038/s41577-020-00446-2 PMC7538181 33024284

[52] Matthews AJ Zheng S DiMenna LJ Chaudhuri J. Regulation of immunoglobulin class-switch recombination: choreography of noncoding transcription, targeted DNA deamination, and long-range DNA repair. Adv Immunol (2014) 122:1–57. doi: 10.1016/B978-0-12-800267-4.00001-8 24507154 PMC4150736

[53] Muramatsu M Kinoshita K Fagarasan S Yamada S Shinkai Y Honjo T. Class switch recombination and hypermutation require activation-induced cytidine deaminase (AID), a potential RNA editing enzyme. Cell (2000) 102(5):553–63. doi: 10.1016/S0092-8674(00)00078-7 11007474

[54] Roco JA Mesin L Binder SC Nefzger C Gonzalez-Figueroa P Canete PF. Class-switch recombination occurs infrequently in germinal centers. Immunity (2019) 51(2):337–350.e337. doi: 10.1016/j.immuni.2019.07.001 31375460 PMC6914312

[55] Reynaud CA Descatoire M Dogan I Huetz F Weller S Weill JC. IgM memory b cells: a mouse/human paradox. Cell Mol Life Sci (2012) 69(10):1625–34. doi: 10.1007/s00018-012-0971-z PMC3337997 22481437

[56] Filippone EJ Farber JL. Humoral immune response and allograft function in kidney transplantation. Am J Kidney Dis (2015) 66(2):337–47. doi: 10.1053/j.ajkd.2015.03.033 25987262

[57] Cabrera-Ortega AA Feinberg D Liang Y Rossa C Jr. Graves DT. The role of forkhead box 1 (FOXO1) in the immune system: Dendritic cells, T cells, b cells, and hematopoietic stem cells. Crit Rev Immunol (2017) 37(1):1–13. doi: 10.1615/CritRevImmunol.2017019636 29431075 PMC6085137

[58] Dominguez-Sola D Victora GD Ying CY Phan RT Saito M Nussenzweig MC. The proto-oncogene MYC is required for selection in the germinal center and cyclic reentry. Nat Immunol (2012) 13(11):1083–91. doi: 10.1038/ni.2428 PMC3711534 23001145

[59] Radtke D Bannard O. Expression of the plasma cell transcriptional regulator blimp-1 by dark zone germinal center b cells during periods of proliferation. Front Immunol (2018) 9:3106. doi: 10.3389/fimmu.2018.03106 30687317 PMC6334666

[60] Pae J Ersching J Castro TBR Schips M Mesin L Allon SJ. Cyclin D3 drives inertial cell cycling in dark zone germinal center b cells. J Exp Med (2021) 218(4):e20201699. doi: 10.1084/jem.20201699 33332554 PMC7754672

[61] Luo W Weisel F Shlomchik MJ. B cell receptor and CD40 signaling are rewired for synergistic induction of the c-myc transcription factor in germinal center b cells. Immunity (2018) 48(2):313–326.e315. doi: 10.1016/j.immuni.2018.01.008 29396161 PMC5821563

[62] Nakagawa R Toboso-Navasa A Schips M Young G Bhaw-Rosun L Llorian-Sopena M. Permissive selection followed by affinity-based proliferation of GC light zone b cells dictates cell fate and ensures clonal breadth. Proc Natl Acad Sci (2021) 118(2):e2016425118. doi: 10.1073/pnas.2016425118 33419925 PMC7812803

[63] Liu B Lin Y Yan J Yao J Liu D Ma W. Affinity-coupled CCL22 promotes positive selection in germinal centres. Nature (2021) 592(7852):133–7. doi: 10.1038/s41586-021-03239-2 33597749

[64] Li X Gadzinsky A Gong L Tong H Calderon V Li Y. Cbl ubiquitin ligases control b cell exit from the germinal-center reaction. Immunity (2018) 48(3):530–541.e536. doi: 10.1016/j.immuni.2018.03.006 29562201

[65] Victora GD Schwickert TA Fooksman DR Kamphorst AO Meyer-Hermann M Dustin ML. Germinal center dynamics revealed by multiphoton microscopy with a photoactivatable fluorescent reporter. Cell (2010) 143(4):592–605. doi: 10.1016/j.cell.2010.10.032 21074050 PMC3035939

[66] Gitlin AD Shulman Z Nussenzweig MC. Clonal selection in the germinal centre by regulated proliferation and hypermutation. Nature (2014) 509(7502):637–40. doi: 10.1038/nature13300 PMC4271732 24805232

[67] Stewart I Radtke D Phillips B McGowan SJ Bannard O. Germinal center b cells replace their antigen receptors in dark zones and fail light zone entry when immunoglobulin gene mutations are damaging. Immunity (2018) 49(3):477–489.e477. doi: 10.1016/j.immuni.2018.08.025 30231983 PMC6162340

[68] Zotos D Quast I Li-Wai-Suen CSN McKenzie CI Robinson MJ Kan A. The concerted change in the distribution of cell cycle phases and zone composition in germinal centers is regulated by IL-21. Nat Commun (2021) 12(1):7160. doi: 10.1038/s41467-021-27477-0 34887406 PMC8660905

[69] Finkin S Hartweger H Oliveira TY Kara EE Nussenzweig MC. Protein amounts of the MYC transcription factor determine germinal center b cell division capacity. Immunity (2019) 51(2):324–336.e325. doi: 10.1016/j.immuni.2019.06.013 31350178 PMC6703930

[70] Razzaghi R Agarwal S Kotlov N Plotnikova O Nomie K Huang DW. Compromised counterselection by FAS creates an aggressive subtype of germinal center lymphoma. J Exp Med. (2021) 218(3) 10.1084/jem.20201173 PMC7694576 33237303

[71] Wilker PR Kohyama M Sandau MM Albring JC Nakagawa O Schwarz JJ. Transcription factor Mef2c is required for b cell proliferation and survival after antigen receptor stimulation. Nat Immunol (2008) 9(6):603–12. doi: 10.1038/ni.1609 PMC2518613 18438409

[72] Tamura T Yanai H Savitsky D Taniguchi T. The IRF family transcription factors in immunity and oncogenesis. Annu Rev Immunol (2008) 26:535–84. doi: 10.1146/annurev.immunol.26.021607.090400 18303999

[73] Ochiai K Maienschein-Cline M Simonetti G Chen J Rosenthal R Brink R. Transcriptional regulation of germinal center b and plasma cell fates by dynamical control of IRF4. Immunity (2013) 38(5):918–29. doi: 10.1016/j.immuni.2013.04.009 PMC3690549 23684984

[74] Bollig N Brüstle A Kellner K Ackermann W Abass E Raifer H. Transcription factor IRF4 determines germinal center formation through follicular T-helper cell differentiation. Proc Natl Acad Sci U S A (2012) 109(22):8664–9. doi: 10.1073/pnas.1205834109 PMC3365194 22552227

[75] Sciammas R Li Y Warmflash A Song Y Dinner AR Singh H. An incoherent regulatory network architecture that orchestrates b cell diversification in response to antigen signaling. Mol Syst Biol (2011) 7:495. doi: 10.1038/msb.2011.25 21613984 PMC3130558

[76] Cumpelik A Heja D Hu Y Varano G Ordikhani F Roberto MP. Dynamic regulation of b cell complement signaling is integral to germinal center responses. Nat Immunol (2021) 22(6):757–68. doi: 10.1038/s41590-021-00926-0 PMC8297556 34031614

[77] Ise W Kurosaki T. Plasma cell differentiation during the germinal center reaction. Immunol Rev (2019) 288(1):64–74. doi: 10.1111/imr.12751 30874351

[78] Minnich M Tagoh H Bönelt P Axelsson E Fischer M Cebolla B. Multifunctional role of the transcription factor blimp-1 in coordinating plasma cell differentiation. Nat Immunol (2016) 17(3):331–43. doi: 10.1038/ni.3349 PMC5790184 26779602

[79] Ding C Chen X Dascani P Hu X Bolli R Zhang H-g. STAT3 signaling in b cells is critical for germinal center maintenance and contributes to the pathogenesis of murine models of lupus. J Immunol (2016) 196(11):4477–86. doi: 10.4049/jimmunol.1502043 PMC4875824 27183592

[80] Carotta S Willis SN Hasbold J Inouye M Pang SHM Emslie D. The transcription factors IRF8 and PU. 1 negatively regulate plasma cell differentiationThe control of plasma cell differentiation. J Exp Med (2014) 211(11):2169–81. doi: 10.1084/jem.20140425 PMC4203955 25288399

[81] Kim SJ Caton M Wang C Khalil M Zhou ZJ Hardin J. Increased IL-12 inhibits b cells' differentiation to germinal center cells and promotes differentiation to short-lived plasmablasts. J Exp Med (2008) 205(10):2437–48. doi: 10.1084/jem.20070731 PMC2556778 18809711

[82] Shinnakasu R Inoue T Kometani K Moriyama S Adachi Y Nakayama M. Regulated selection of germinal-center cells into the memory b cell compartment. Nat Immunol (2016) 17(7):861–9. doi: 10.1038/ni.3460 27158841

[83] Chiu Y-K Lin I-Y Su S-T Wang K-H Yang S-Y Tsai D-Y. Transcription factor ABF-1 suppresses plasma cell differentiation but facilitates memory b cell formation. J Immunol (2014) 193(5):2207–17. doi: 10.4049/jimmunol.1400411 25070843

[84] Scheeren FA Naspetti M Diehl S Schotte R Nagasawa M Wijnands E. STAT5 regulates the self-renewal capacity and differentiation of human memory b cells and controls bcl-6 expression. Nat Immunol (2005) 6(3):303–13. doi: 10.1038/ni1172 15711548

[85] Scharer CD Barwick BG Guo M Bally APR Boss JM. Plasma cell differentiation is controlled by multiple cell division-coupled epigenetic programs. Nat Commun (2018) 9(1):1698. doi: 10.1038/s41467-018-04125-8 29703886 PMC5923265

[86] Su IH Basavaraj A Krutchinsky AN Hobert O Ullrich A Chait BT. Ezh2 controls b cell development through histone H3 methylation and igh rearrangement. Nat Immunol (2003) 4(2):124–31. doi: 10.1038/ni876 12496962

[87] Barwick BG Scharer CD Martinez RJ Price MJ Wein AN Haines RR. B cell activation and plasma cell differentiation are inhibited by *de novo* DNA methylation. Nat Commun (2018) 9(1):1900. doi: 10.1038/s41467-018-04234-4 29765016 PMC5953949

[88] Pitzalis C Jones GW Bombardieri M Jones SA. Ectopic lymphoid-like structures in infection, cancer and autoimmunity. Nat Rev Immunol (2014) 14(7):447–62. doi: 10.1038/nri3700 24948366

[89] MacLennan IC Toellner KM Cunningham AF Serre K Sze DM Zúñiga E. Extrafollicular antibody responses. Immunol Rev (2003) 194:8–18. doi: 10.1034/j.1600-065X.2003.00058.x 12846803

[90] Purtha WE Tedder TF Johnson S Bhattacharya D Diamond MS. Memory b cells, but not long-lived plasma cells, possess antigen specificities for viral escape mutants. J Exp Med (2011) 208(13):2599–606. doi: 10.1084/jem.20110740 PMC3244041 22162833

[91] Armitage RJ Fanslow WC Strockbine L Sato TA Clifford KN Macduff BM. Molecular and biological characterization of a murine ligand for CD40. Nature (1992) 357(6373):80–2. doi: 10.1038/357080a0 1374165

[92] Miles B Connick E. Control of the germinal center by follicular regulatory T cells during infection. Front Immunol (2018) 9:2704. doi: 10.3389/fimmu.2018.02704 30524440 PMC6256122

[93] Linterman MA Pierson W Lee SK Kallies A Kawamoto S Rayner TF. Foxp3+ follicular regulatory T cells control the germinal center response. Nat Med (2011) 17(8):975–82. doi: 10.1038/nm.2425 PMC3182542 21785433

[94] Xu B Wang S Zhou M Huang Y Fu R Guo C. The ratio of circulating follicular T helper cell to follicular T regulatory cell is correlated with disease activity in systemic lupus erythematosus. Clin Immunol (2017) 183:46–53. doi: 10.1016/j.clim.2017.07.004 28709914 PMC5673570

[95] Zhao S Ding J Wang S Li C Guo P Zhang M. Decreased expression of circulating aire and increased Tfh/Tfr cells in myasthenia gravis patients. Biosci Rep (2018) 38(6):BSR20180096. doi: 10.1042/BSR20180096 29773681 PMC6239276

[96] Yan L Li Y Li Y Wu X Wang X Wang L. Increased circulating tfh to tfr ratio in chronic renal allograft dysfunction: a pilot study. BMC Immunol (2019) 20(1):26. doi: 10.1186/s12865-019-0308-x 31382877 PMC6683539

[97] de Graav GN Dieterich M Hesselink DA Boer K Clahsen-van Groningen MC Kraaijeveld R. Follicular T helper cells and humoral reactivity in kidney transplant patients. Clin Exp Immunol (2015) 180(2):329–40. doi: 10.1111/cei.12576 PMC4408167 25557528

[98] de Leur K Clahsen-van Groningen MC van den Bosch TPP de Graav GN Hesselink DA Samsom JN. Characterization of ectopic lymphoid structures in different types of acute renal allograft rejection. Clin Exp Immunol (2018) 192(2):224–32. doi: 10.1111/cei.13099 PMC5904712 29319177

[99] Alsughayyir J Chhabra M Qureshi MS Mallik M Ali JM Gamper I. Relative frequencies of alloantigen-specific helper CD4 T cells and b cells determine mode of antibody-mediated allograft rejection. Front Immunol (2018) 9:3039. doi: 10.3389/fimmu.2018.03039 30740108 PMC6357941

[100] Louis K Fadakar P Macedo C Yamada M Lucas M Gu X. Concomitant loss of regulatory T and b cells is a distinguishing immune feature of antibody-mediated rejection in kidney transplantation. Kidney Int (2022) 101(5):1003–16. doi: 10.1016/j.kint.2021.12.027 PMC9038633 35090879

[101] Niu Q Mendoza Rojas A Dieterich M Roelen DL Clahsen-van Groningen MC Wang L. Immunosuppression has long-lasting effects on circulating follicular regulatory T cells in kidney transplant recipients. Front Immunol (1972) 2020:11. doi: 10.3389/fimmu.2020.01972 PMC7483930 32983131

[102] Macedo C Walters JT Orkis EA Isse K Elinoff BD Fedorek SP. Long-term effects of alemtuzumab on regulatory and memory T-cell subsets in kidney transplantation. Transplantation (2012) 93(8):813–21. doi: 10.1097/TP.0b013e318247a717 PMC3323763 22343334

[103] Noureldeen T Albekioni Z Machado L Muddana N Marcus RJ Hussain SM. Alemtuzumab induction and antibody-mediated rejection in kidney transplantation. Transplant Proc (2014) 46(10):3405–7. doi: 10.1016/j.transproceed.2014.08.037 25498060

[104] Budde K Prashar R Haller H Rial MC Kamar N Agarwal A. Conversion from calcineurin inhibitor– to belatacept-based maintenance immunosuppression in renal transplant recipients: A randomized phase 3b trial. J Am Soc Nephrology (2021) 32(12):3252–64. doi: 10.1681/ASN.2021050628 PMC8638403 34706967

[105] Vincenti F Rostaing L Grinyo J Rice K Steinberg S Gaite L. Belatacept and long-term outcomes in kidney transplantation. N Engl J Med (2016) 374(4):333–43. doi: 10.1056/NEJMoa1506027 26816011

[106] Vincenti F Charpentier B Vanrenterghem Y Rostaing L Bresnahan B Darji P. A phase III study of belatacept-based immunosuppression regimens versus cyclosporine in renal transplant recipients (BENEFIT study). Am J Transplant (2010) 10(3):535–46. doi: 10.1111/j.1600-6143.2009.03005.x 20415897

[107] Schroder PM Schmitz R Fitch ZW Ezekian B Yoon J Choi AY. Preoperative carfilzomib and lulizumab based desensitization prolongs graft survival in a sensitized non-human primate model. Kidney Int (2021) 99(1):161–72. doi: 10.1016/j.kint.2020.08.020 PMC7785689 32898569

[108] Ezekian B Schroder PM Mulvihill MS Barbas A Collins B Freischlag K. Pretransplant desensitization with costimulation blockade and proteasome inhibitor reduces DSA and delays antibody-mediated rejection in highly sensitized nonhuman primate kidney transplant recipients. J Am Soc Nephrol (2019) 30(12):2399–411. doi: 10.1681/ASN.2019030304 PMC6900797 31658991

[109] Carfilzomib and belatacept for desensitization. Available at: https://ClinicalTrials.gov/show/NCT05017545.

[110] Daratumumab and belatacept for desensitization. Available at: https://ClinicalTrials.gov/show/NCT04827979.

[111] Conlon TM Cole JL Motallebzadeh R Harper I Callaghan CJ Bolton EM. Unlinked memory helper responses promote long-lasting humoral alloimmunity. J Immunol (2012) 189(12):5703–12. doi: 10.4049/jimmunol.1202257 PMC3788591 23162131

[112] Gorbacheva V Fan R Wang X Baldwin WM 3rd Fairchild RL Valujskikh A. IFN-γ production by memory helper T cells is required for CD40-independent alloantibody responses. J Immunol (2015) 194(3):1347–56. doi: 10.4049/jimmunol.1401573 PMC4297685 25548230

[113] Lavinder JJ Horton AP Georgiou G Ippolito GC. Next-generation sequencing and protein mass spectrometry for the comprehensive analysis of human cellular and serum antibody repertoires. Curr Opin Chem Biol (2015) 24:112–20. doi: 10.1016/j.cbpa.2014.11.007 25461729

[114] Chen M Hong MJ Sun H Wang L Shi X Gilbert BE. Essential role for autophagy in the maintenance of immunological memory against influenza infection. Nat Med (2014) 20(5):503–10. doi: 10.1038/nm.3521 PMC4066663 24747745

[115] Chen M Kodali S Jang A Kuai L Wang J. Requirement for autophagy in the long-term persistence but not initial formation of memory b cells. J Immunol (2015) 194(6):2607–15. doi: 10.4049/jimmunol.1403001 PMC4355050 25672753

[116] Kodali S Li M Budai MM Chen M Wang J. Protection of quiescence and longevity of IgG memory b cells by mitochondrial autophagy. J Immunol (2022) 208(5):1085–98. doi: 10.4049/jimmunol.2100969 PMC8887795 35101890

[117] Wu J Zhang H Shi X Xiao X Fan Y Minze LJ. Ablation of transcription factor IRF4 promotes transplant acceptance by driving allogenic CD4(+) T cell dysfunction. Immunity (2017) 47(6):1114–1128.e1116. doi: 10.1016/j.immuni.2017.11.003 29221730 PMC5759774

[118] Wang G Zou D Wang Y Gonzalez NM Yi SG Li XC. IRF4 ablation in b cells abrogates allogeneic b cell responses and prevents chronic transplant rejection. J Heart Lung Transplant (2021) 40(10):1122–32. doi: 10.1016/j.healun.2021.06.008 PMC8464533 34253454

